# Barriers and Facilitators to Cardiovascular Disease Prevention Following Hypertensive Disorders of Pregnancy in Primary Care: Cross-Sectional Surveys

**DOI:** 10.3390/nu15173817

**Published:** 2023-08-31

**Authors:** Kaylee Slater, Rachael Taylor, Karen McLaughlin, Craig Pennell, Clare Collins, Melinda Hutchesson

**Affiliations:** 1 School of Health Sciences, College of Health, Medicine and Wellbeing, University of Newcastle, Callaghan, NSW 2308, Australia; kaylee.slater@uon.edu.au (K.S.); rachael.taylor@newcastle.edu.au (R.T.); clare.collins@newcastle.edu.au (C.C.); 2 Food and Nutrition Research Program, Hunter Medical Research Institute, Lot 1, Kookaburra Circuit, New Lambton Heights, NSW 2305, Australia; 3 School of Nursing and Midwifery, College of Health, Medicine and Wellbeing, University of Newcastle, Callaghan, NSW 2308, Australia; karen.mclaughlin@newcastle.edu.au; 4 School of Medicine and Public Health, College of Health, Medicine and Wellbeing, University of Newcastle, Callaghan, NSW 2308, Australia; craig.pennell@newcastle.edu.au

**Keywords:** preeclampsia, gestational hypertension, primary care, general practice, general practitioners, cardiovascular disease, cardiovascular risk, preventative health, prevention

## Abstract

Women with a history of hypertensive disorders of pregnancy (HDP) have an increased risk of cardiovascular disease (CVD). Guidelines recommend that women diagnosed with HDP should be advised of their increased CVD risk, have regular blood pressure monitoring by their general practitioner (GP), and adopt healthy lifestyle behaviours. However, within Australia, the current practice in primary health care is unknown. The aim of this study was to describe current practices, barriers, and facilitators to the provision of CVD preventative services for women after HDP in the primary care setting and to identify potential strategies to support GPs in providing recommended care. Separate cross-sectional online surveys were undertaken with 35 GPs and 105 women with a history of HDP. Surveys included both closed- and open-ended questions. Closed-ended questions were analysed using basic descriptive statistics, and open-ended questions were themed and tallied. The survey of GPs revealed that GPs are more likely to assess traditional CVD risk markers than lifestyle risk factors or HDP history. GPs identified a lack of resources and skills as barriers to providing CVD preventative care post-HDP. The survey with women after HDP revealed that women with a history of HDP are more likely to be assessed for blood pressure than lifestyle CVD risk factors, and that the women’s barriers to obtaining care included difficulty obtaining an appointment and time required for attending appointments. Strategies to improve CVD preventative care were consistent between surveys, where 70% of GPs and 59% of women chose ‘increasing women’s awareness of increased CVD risk’ and 67% of GPs and 55% of women chose ‘improving communication between hospitals and primary care’ as their preferred strategies. While the findings suggest that women with a history of HDP are receiving advice consistent with guidelines for traditional CVD risk markers, such as blood pressure, they are less likely to receive CVD preventative care for lifestyle or female-specific CVD risk factors.

## 1. Introduction

Hypertensive disorders of pregnancy (HDP), which affect 8–10% of pregnancies worldwide, encompass a group of diseases, including preeclampsia and gestational hypertension [1,2]. Women who develop HDP have a two- to four-fold increased risk of developing cardiovascular disease (CVD) within 10 years [1,3,4]. Numerous systematic reviews have demonstrated a relationship between HDP and CVD [4,5,6,7,8,9,10]. Most recently, in a 2020 meta-analysis of 73 studies with over 13-million participants, Wu et al. observed the relative risks of CVD were higher amongst women with a history of HDP compared to those with normotensive pregnancies, including hypertension (Relative Risk (RR): 3.16, 95% confidence interval (CI) 2.74–3.64), heart failure (RR: 2.87, 95% CI 2.14–3.85), stroke (RR: 1.72, 95% CI 1.50–1.97), coronary heart disease (RR: 1.66, 95% CI 1.49–1.84), and peripheral vascular disease (RR: 1.60, 95% CI 1.29–2.00) [4]. It is suggested that after a preeclamptic pregnancy specifically, women exhibit a subclinical pathology shared with metabolic syndrome and obesity, increased vascular resistance, insulin resistance, and hyperlipidaemia [1]. Therefore, it is important that appropriate management of CVD risk factors occurs soon after pregnancy for the prevention of CVD in later life.

CVD prevention involves assessment of traditional risk factors, including risk markers (blood pressure, plasma lipids, and blood glucose levels), lifestyle risk factors (diet, physical activity, smoking, alcohol consumption, weight management, sleep, and mental health), as well as female-specific risk factors, such as obstetric history [11]. Internationally, including in Australia, clinical guidelines recommend that post-HDP women are advised of their increased CVD risk, encouraged to have regular blood pressure monitoring by their general practitioner (GP), and adopt healthy lifestyle behaviours [3,12]. The International Society for the Study of Hypertension in Pregnancy (ISSHP) guidelines, among others, suggest that women after HDP should be advised of their increased CVD risk [3,12,13]. Current observational research suggests that healthcare professionals (HCPs) in developed countries involved in postpartum care are aware of the increased risk of CVD post-HDP [14,15,16,17]. However, women are unaware of their increased risk of CVD and report receiving little preventative care post-HDP. A scoping review published in 2019 examining women’s knowledge of CVD after HDP reported that in six out of seven included studies women had either limited or no knowledge of their increased risk of CVD [16]. 

During the postpartum period, there is a unique opportunity for assessment of CVD risk and appropriate intervention for women post-HDP. In Australia, it is recommended that women attend a six-week postpartum check-up with their GP. The purpose of this check-up is to assess the new mother’s physical health and recovery post-childbirth as well as discuss their emotional health and well-being [3,18]. In Ref. [18], for women that have experienced HDP, this appointment gives their GP the opportunity to review their medication and assess their blood pressure [3]. 

National level data on uptake of these appointments in Australia are not available; however, findings from a longitudinal needs analysis of breastfeeding behaviours in a Queensland regional town suggested that by 12 weeks postpartum most mothers had visited a GP [19]. Similarly, primary care records of over 34,000 women in England from 2015 to 2018 indicated that 89% of women visited their GP 12 weeks after child birth, and 62% saw a GP for a six-week postpartum assessment [20]. 

A scoping review of 12 studies conducted between 2005 and 2019, including 402 women and 1215 health professionals from seven countries, revealed that despite regular follow-up of women post-pregnancy current practices for CVD prevention after HDP do not align with clinical guidelines; however, it is unclear why this is occurring [16]. Such knowledge will increase awareness of facilitators that are enhancing current health practices, as well as the gaps in the current health system, and identify potential strategies that may alleviate these gaps [21]. Therefore, the aim of this study was to explore current practices, barriers, and facilitators to engaging in CVD prevention from both GPs and women after HDP in a large local health district covering regional, rural, and remote communities in New South Wales (NSW), Australia, as well as potential strategies that would support GPs to provide recommended care.

## 2. Materials and Methods

### 2.1. Study Design and Participants 

The current study was a primary data analysis of two cross-sectional surveys. The Checklist for Reporting Results of Internet E-Surveys (CHERRIES) was used for reporting the survey results [22], and the Strengthening the Reporting of Observational Studies in Epidemiology (STROBE) Statement was applied for the reporting of this observational study [23]. Ethics approval was received from the University of Newcastle Human Research Ethics Committee H-2021-0296 for the GP survey and H-2021-0415 for the women after HDP survey. 

Participants were GPs practicing within the Hunter New England Local Health District (HNELHD) in NSW, Australia, and Australian women (>18 years old) living in the HNELHD with a history of HDP in one or more pregnancy in the previous five years at survey recruitment (2017 to 2022). HNELHD is the only district in NSW with a major metropolitan centre, a mix of several large regional centres, and many smaller rural and remote communities within its borders. People living in rural and remote communities generally experience less access to health services and consequently poorer health outcomes [24]. Therefore, it is important to understand current healthcare practices in a district that covers a range of diverse communities. 

### 2.2. Recruitment 

#### 2.2.1. GP Survey

Recruitment took place in October 2021, where The Australian Medical Publishing Company (AMPCo) emailed a sample of 693 GPs using postcodes in HNELHD. AMPCO database is a comprehensive catalogue of medical practitioners, including GPs in Australia, that has been used previously to recruit GPs [25]. GPs were sent an email to participate by AMPCo on behalf of the researchers containing a weblink to an information statement and the online survey. A follow-up email was sent two weeks after the initial email. Digital recruitment material was also distributed on the Royal Australia College of General Practitioners’ NSW division Facebook page and via an email newsletter distributed to the Hunter Postgraduate Medical Institute, an organisation that provides professional development for health professionals in the HNELHD region. All participating GPs received a $35 e-gift card as remuneration of their time.

#### 2.2.2. History of HDP Survey

Recruitment took place from February to July 2022. Australian women with a history of HDP were informed of the survey via several recruitment strategies. Recruitment took place through social media (Facebook and Instagram). Posts were made, with permission of moderators, to five HDP-focused Facebook groups (>15,000 members) and HNELHD-specific groups targeting parents (>20,000 members). Recruitment material was also shared with not-for-profit organisations targeting women that experienced pregnancy complications (Australasian Birth Trauma Association and the Centre of Perinatal Excellence) and experts in the areas of women’s health, preconception, and pregnancy, requesting they share recruitment materials on their social media accounts, such as Instagram, Facebook, and Twitter. Researchers paid a fee to the Centre of Perinatal Excellence to share recruitment material on their social media pages. The survey itself recruited women Australia-wide; however, participants included in this study were only living in the HNELHD and were identified via self-reported postcodes. Data, including the full sample of women Australia-wide, will be published elsewhere. All participants who returned a survey were given the opportunity to go into a prize draw to win one of 10 $200 e-gift cards as a token of appreciation for participating. 

### 2.3. Data Collection 

All data were collected and managed using REDCap (Research Electronic Data Capture) electronic data capture tools, version 12.5.5 by Vanderbilt University, hosted at the University of Newcastle, Australia. REDCap is a secure, web-based software platform designed to support data capture for research studies [26,27].

#### 2.3.1. GP Survey

The GP survey included 17 questions, including 14 closed- and three open-ended questions. This survey captured demographic characteristics of GPs (e.g., gender identity, age, postcode of main workplace, description of main practice and years practicing as a GP). Current practice of CVD preventive actions with women, such as knowledge and use of relevant guidelines and assessment and management of lifestyle risk factors (e.g., diet), CVD risk markers (e.g., blood pressure), and female-specific risk factors (e.g., obstetric history) were asked using closed-ended questions and measured using a 5-point Likert scale on components included in respective CVD prevention guidelines [3,12]. Perceived barriers and enablers to CVD prevention with women post-HDP were explored using the Theoretical Domains Framework (TDF) [28] and informed by previous research by Roth et al. [29]. The TDF is a validated theoretical framework that specifies a range of actions for behaviour change in health professionals [30]. Barriers and enablers were presented as statements, where GPs graded their agreement with each statement on a five-point Likert scale ranging from 1 = strongly disagree to 5 = strongly agree. In the survey, GPs were also given the opportunity to elaborate on additional barriers experienced when managing CVD risk post-HDP. Potential strategies to support them in implementing recommendations from clinical guidelines were also presented as statements where GPs could rank their top three preferred strategies. These strategies were derived from previous research of CVD prevention for women after HDP [16,29] and expert opinion of the research team. An open-ended question allowed GPs to elaborate on the strategies they had chosen to be prioritised and to suggest additional strategies for improving CVD preventative care provided to women post-HDP.

#### 2.3.2. Survey for Women with a History of HDP

The survey for women with a history of HDP included 31 questions, including 30 closed-ended questions and one open-ended question. Key sociodemographic characteristics of women following HDP were captured via closed-ended questions (e.g., age, education level, postcode, pregnancy history). Current practice of CVD prevention services provided by their respective HCPs since their HDP diagnosis included closed-ended questions (e.g., asking which health professionals provided advice for CVD risk markers and lifestyle CVD risk factors). Perceived barriers and enablers were captured using a range of closed-ended questions (e.g., asking what factors discourage participants from seeing GPs, as well as providing a 5-point Likert scale asking participants how supported they feel by their GP on a variety of lifestyle CVD risk factors). Lastly, potential strategies to support them in obtaining recommended CVD care after HDP were presented as statements where participants could rank their top three preferred strategies. These strategies were consistent with the GP survey. An open-ended question allowed women to elaborate on additional barriers they face when obtaining CVD preventive care from GPs after HDP and suggest additional strategies to improve the accessibility of this care. 

### 2.4. Data Analysis 

Data analysis for closed-ended questions was conducted using STATA statistical software (v. 16) [31] and was analysed using basic descriptive statistics, whereas open-ended questions were analysed via thematic analysis. For the GP survey, where surveys were incomplete, the data that were recorded for ≥50% of the questions were still included in the final analysis (*n* = 4); however, in the history of HDP survey, incomplete data were excluded from the final analysis. This was required because in the GP survey the demographic characteristics questions were at the beginning of the survey, whereas for the history of HDP survey, these questions were asked at the end to not deter participants from completing the survey. Therefore, in the history of HDP survey, to include only participants from HNELHD, only those who completed the demographic characteristics questions were included in the data analysis. Thematic analyses were applied to two open-ended questions in the GP survey: “what other barriers (if any) do you experience in the assessment and management of lifestyle risk factors for women who have experienced HDP?” and “what other strategies, if any, would assist or support GPs in the assessment and/or management of lifestyle risk factors following HDP?” In the history of the HDP survey, thematic analysis was applied to the following question: “Are there any other strategies you believe GPs could implement to ensure they are providing the best support to you for managing your heart health?” Answers from these questions were labelled and coded, and the codes were combined and categorised into overarching themes, supported by participant quotes. Additional analysis was undertaken to explore differences in current practice, barriers, and facilitators to engaging in CVD prevention and potential strategies that may improve CVD prevention by sociodemographic characteristics (age and geographical location for both GPs and women after HDP and socioeconomic status for only women after HDP). To determine geographical location, postcodes provided by participants were characterised as either Greater Newcastle (Metropolitan) or all other areas of HNELHD (regional/rural). Socioeconomic status for the women after HDP was classified based on a self-reported postcode using the Socio-Economic Indexes for Areas (SEIFA) indexes [32]. The SEIFA indexes rank areas in Australia according to their relative socio-economic advantage and disadvantage, which is based on key dimensions, including income, education, employment, occupation, and housing recorded in the Census data [32]. Postcodes were matched using the Postal Area Index of Relative Socioeconomic Advantage and Disadvantage 2021. Continuous data were assessed for normality using the Shapiro–Wilks test of normality. Analysis of variance (one-way ANOVA) tests were used to assess continuous variables (age) and χ^2^ tests for categorical variables (geographical location and SES) for significant differences. *p*-values of <0.05 were considered statistically significant. 

## 3. Results

### 3.1. Participants 

Overall, there were 35 participants in the GP survey and 105 in the history of HDP survey. Table 1 describes the characteristics of GPs and women with a history of HDP in the study. The GPs were mostly female (*n* = 31, 86.1%) and worked in large group practices (≥6 clinicians) (*n* = 23, 63.9%). In this sample, 94% of GPs practiced in metropolitan or regional areas (Greater Newcastle, Lower Hunter/Hunter Valley, Lower Mid North Coast). This was similar for the women, where 95% resided in urban areas (Greater Newcastle, Lower Hunter/Hunter Valley). This was grouped as metropolitan (*n* = 31, 60%) and regional/rural (*n* = 14, 40%) practice areas. The women in the sample were mostly diagnosed with HDP from 2020 onwards (*n* = 68, 64.8%), and a majority had preeclampsia (*n* = 65, 61.9%), whereas a smaller number had either gestational hypertension (*n* = 37, 35.2%) and/or chronic hypertension (*n* = 11, 10.5%). Additionally, >90% of women were born in Australia/New Zealand, 56% had tertiary education, and >56% had annual household incomes above AUD$91,000. Women were also grouped by the HNELHD residential area as metropolitan (*n* = 70, 66.7%) and regional/rural (*n* = 35, 33.3%).

### 3.2. Current Practice Regarding the Provision of CVD Preventative Services Post-HDP 

The guidelines for management of absolute CVD risk by the Initiative of the National Vascular Disease Prevention Alliance were used by more than half of the GPs (*n* = 19, 55.9%), whereas 8.8% of GPs (*n* = 3) reported regularly using the ISSHP guidelines and 23.5% the Society of Obstetric Medicine Australia and New Zealand (SOMANZ) guidelines for the management of HDP. There were no significant differences in GPs use or knowledge of guidelines by age or geographical location. 

Table 2 reports the frequency with which GPs discuss traditional and female-specific CVD risk factors with women post-HDP. CVD risk markers, blood pressure (*n* = 33, 97.1% of GPs assessed this bi-annually), plasma lipids (*n* = 25, 75.3% of GPs assessed this annually), and blood glucose/insulin (*n* = 24, 70.5% of GPs assessed this annually) were mostly assessed by GPs. Ninety-four percent of GPs often/always assessed women with a history of HDP for smoking status, and 83% always/often assessed alcohol consumption and body weight. When assessing medical history, female-specific CVD risk factors, such as preeclampsia, gestational hypertension, and gestational diabetes, were always/often assessed by less than 70% of GPs. Traditional medical risk factors, such as family history of heart disease, high blood pressure, high blood glucose levels, and high lipid levels, were always/often assessed by more than 90% of GPs. Most GPs reported assessing preterm birth (*n* = 24, 72.7%), small for gestational age (*n* = 24, 72.7%), and polycystic ovarian syndrome (*n* = 18, 54.6%) either sometimes, occasionally, or not at all. There were no significant differences in GPs’ current practice of CVD prevention with women after HDP by age or geographical location. 

Table 3 presents women’s awareness of CVD risk factors and CVD risk assessment by health professionals. Of the women with a history of HDP, 17% (*n* = 29) were aware of their increased CVD risk before participating in this survey, and 15% of women (*n* = 27) were advised to visit their GP during the postpartum period. For CVD risk markers, 83% of women with a history of HDP had their blood pressure assessed, and 72% had their obstetric history assessed, which was predominantly by their GP. Less than 50% of women with a history of HDP had their blood lipids and blood glucose levels assessed. Whereas for lifestyle risk factors, 80% many women reported that a health professional assessed and managed their mental health (80%), physical activity levels (57%), and dietary intake (51%), and this was also predominantly by their GP. There were no significant differences in women’s awareness of CVD risk factors or CVD risk assessment by health professionals by age, geographical location, or SES.

### 3.3. Barriers and Facilitators to the Provision or the Obtainment of CVD Preventative Services

Table 4 reports GPs’ agreement with statements from each domain of the TDF relating to CVD prevention for women post-HDP. Survey results indicate that GPs in this sample agree that managing CVD lifestyle risk factors in women post-HDP is their role (knowledge, mean 4.6). However, fewer GPs believe that they have the necessary skills to perform this role (skills, mean 3.4). GPs also consistently agreed that women post-HDP would benefit from receiving advice for managing lifestyle CVD risk factors (belief about consequences, mean 4.6). However, many neither agreed nor disagreed with having a detailed plan of how they would provide this care (environmental context and resources, mean 3.1). There were statistically significant differences among GPs from the metropolitan compared with the regional/rural practice areas when it came to agreement on three of the 12 barriers. GPs in the regional/rural compared to the metropolitan HNELHD were more likely to agree (agree or strongly agree) that they had skills (*n* = 11, 79% and *n* = 5, 24%, *p* = 0.04); memory, attention, and decision processes (*n* = 7, 50% and *n* = 1, 5%, *p* = 0.02); and behavioural regulation (*n* = 10, 71% and *n* = 4, 19%, *p* = 0.02) to provide CVD prevention to women after HDP. There were no significant differences in GPs barriers or facilitators to CVD prevention with women after HDP by age.

GPs were asked whether they experience other barriers for assessing and/or managing CVD lifestyle risk factors for women post-HDP. Table 5 reports the four main themes identified from the survey responses were lack of time, lack of knowledge and training, lack of patient motivation, and lack of awareness of patients’ obstetric history. Lack of time and patients’ lack of motivation to engage in lifestyle CVD prevention were the most common themes reported. Women with a history of HDP were not asked this question in the survey.

Table 6 reports the barriers women face when accessing health care from a GP. The main barriers that women faced when seeing the GP were difficulty obtaining an appointment (*n* = 59, 56.2%), the time it took to see the GP (*n* = 38, 36.2%), and the cost involved (*n* = 36, 34.3%). Notably, 19% of women did not perceive there to be any barriers for accessing health care from GPs (*n* = 20, 19.1%). There were statistically significant differences in the number of women who thought cost was a barrier to accessing health services from GPs when comparing those living in the metropolitan (*n* = 29, 41.4%) compared with the regional/rural (*n* = 7, 20.0%) HNELHD (*p* = 0.03). There were no significant differences in women’s reported barriers to accessing health care for a GP by age and SES. Participants also reported other barriers faced when assessing CVD prevention care from GPs (Table 6). 

Table 7 demonstrated the level of support women with a history of HDP feel from their GP in regard to making changes to CVD lifestyle risk factors. More than 50% of women felt supported (very/somewhat supported) by their GP for stress/mental health support (*n* = 72, 68.6%), diet (*n* = 57, 54.3%), and physical activity (*n* = 55, 52.4%). More than 50% of women felt that obtaining support for smoking and alcohol consumption was not applicable to them, but among those who did, 20% and 25% felt supported, respectively. There were statistically significant differences in the level of support women felt from their GP in managing stress/mental health when comparing those living in the metropolitan (*n* = 43, 61.4% felt very/somewhat supported) compared with the regional/rural (*n* = 29, 82.9% felt very/somewhat supporter) (*p* = 0.03) areas, but not for any other lifestyle CVD risk factors. There were no significant differences in women’s perceived level of support from their GP for age and SES.

### 3.4. Potential Strategies for the Provision of CVD Preventative Services after HDP in Primary Care

GPs and women with a history of HDP were presented with eight potential strategies, each to support the provision of CVD preventative services for women after HDP and were asked to rate their top three preferred strategies (Table 8). The GP’s two highest-rated strategies were ‘improve communication to women of HDP and long-term effects’ (*n* = 23, 69.7%) and ‘improvements in communication from hospitals to primary care’ (*n* = 22, 66.7%). The priority strategies that women most frequently included in their top three included communicating the long-term effects after HDP (*n* = 62, 59.1%), improved communication between health professionals (*n* = 58, 55.2%), and referrals to other health professionals for CVD preventative care (*n* = 52, 49.5%). There were no significant differences in potential strategies to improve CVD prevention for women after HDP for GPs, women by age and geographical location, and women by SES.

Women in the history of HDP survey were also asked if they had additional strategies to suggest (Table 9). Written answers were broken down into three main themes: more support and acknowledgement of obstetric history, attending more than one postpartum appointment and/or a heart heath specific appointment, and being sent regular reminders to follow up with their GP. A similar open-ended question was asked to the GPs; however, the answers were reiterations of the strategies already presented above and, therefore, are not uniquely presented within this analysis.

## 4. Discussion

The current study explores current practices, barriers, and facilitators to providing CVD preventative care to women following HDP, as well as potential strategies to improve the care provided to women after HDP among GPs and women with a history of HDP from regional, rural, and remote communities in NSW, Australia. Results indicate that GPs are assessing women for traditional CVD risk factors, such as blood pressure; however, they are less likely to assess female-specific risk factors, such as HDP or lifestyle CVD risk factors, such as diet and physical activity, as part of routine CVD assessment in women. GPs and women also identified several key barriers to the provision of CVD preventative services within primary care (e.g., lack of skills and training, insufficient environmental support, and resources for CVD prevention post-HDP, and difficulty obtaining an appointment). However, the barriers identified did not align with strategies that they believed would improve the provision of CVD preventive care, such as improving communication of CVD risk to women after HDP, improving communication between HCPs, and referrals to other HCPs for CVD care. 

Clinical guidelines suggest that women’s CVD risk should be frequently monitored post-HDP [3,12]. The ISSHP and SOMANZ guidelines place the responsibility on GPs to regularly monitor blood pressure of women post-HDP, including a five-year check of plasma lipids and blood glucose levels, and encourage women to adopt a healthy lifestyle. Both the current surveys suggest that GPs are measuring women’s blood pressure consistent with the guidelines; however, there were discrepancies between the two surveys for monitoring blood lipids and blood glucose levels, highlighting the differences in perceived care amongst GPs and women with a history of HDP. Similar results were observed in a 2018 survey of 127 Australian women with a recent history of preeclampsia (≤2 years), where Hutchesson et al. asked participants whether they had received advice or screening as per the SOMANZ guidelines [35]. Most women (94.5%) had their blood pressure measured, although only 40.9% had their cholesterol or glucose checked [35]. Although findings suggest that GPs are measuring blood pressure, it also appears that information about ongoing CVD risk may not be communicated to the women by their GP. Additionally, GPs in the current sample reported assessing traditional CVD risk factors consistent with ISSHP and SOMANZ guidelines, it appears that they were less likely to assess lifestyle risk factors and female-specific CVD risk factors, such as HDP. Similarly, in a USA-based online cross-sectional survey (*n* = 53), Gogineni et al. revealed that 36% of internal medicine and 50% of family medicine physicians monitor every woman for traditional CVD risk factors, but the majority did not consider adverse pregnancy outcomes, including HDP, when assessing CVD risk [36]. Women in the current sample reiterated that GPs were less likely to ask about lifestyle CVD risk factors. Similarly, previous research indicated that GPs have more confidence in managing traditional CVD risk factors and less for managing lifestyle CVD risk factors [37]. 

In a recent qualitative interpretive study, Ray et al. described the experiences of Australian women in receiving information about pregnancy complications from HCPs [38]. Ray et al. reported that women found communication from their HCPs distressing, and had concerns over the delay in information regarding their care [38]. In contrast, while the current study investigates care after HDP instead of during pregnancy, a majority of women in this sample were satisfied by the care provided by their GP and felt supported when it came to managing stress and mental health, specifically amongst women living in regional/rural communities in the HNELHD. In the current sample, most GPs agreed that providing women with CVD preventative care post-HDP was their responsibility. Barriers to providing this care included insufficient training, workplace support, and lack of resources, and interestingly, more GPs practicing in the metropolitan HNELHD felt that they did not have the skills to manage CVD prevention for women after HDP. Ray et al. argued that the current medical system of antenatal care for women with pregnancy complications does not meet current Australian guidelines [38]. However, the current study findings suggest CVD care for traditional risk factors (e.g., blood pressure) after HDP are consistent with the SOMANZ guidelines. Although, both the GPs in this sample and women with a history of HDP were less likely to report that advice regarding healthy lifestyles was provided, which may reflect GPs’ lack of training, support, and resources related to lifestyle CVD risk assessment and management. 

Despite GPs’ reporting knowledge of elevated CVD risk post-HDP, the majority of women with a history of HDP are unaware of their increased risk of CVD, which is also evident within this study [16]. A highly ranked strategy by both GPs and women with a history of HDP was to improve the knowledge of women regarding their increased CVD risk after HDP. A similar qualitative study of 13 women with a history of HDP identified that when transitioning from hospital to primary care women also wanted to receive information about their long-term medical follow-up [39]. Without the knowledge of their long-term CVD risks, women would be unaware of a need to inform their GP of their obstetric history, specifically pregnancy complications, like HDP. One of the highest ranked strategies amongst GPs and women with a history of HDP was improving communication between primary care and the hospital systems, which could address this, as GPs would already be aware of women’s obstetric history. Comparably, it has been acknowledged in previous research that communication between hospitals and primary care is infrequent [6,40], with one research study also identifying that hospital discharge summaries often lacked important information, such as diagnostic test results and follow-up plans [40]. Women with a history of HDP in the current sample also ranked improving the referral process to other HCPs highly, which would allow them to obtain necessary advice from a multidisciplinary team. Roth et al. stated that HCPs wanted to improve health literacy amongst women after HDP, specifically needing suitable and supportive materials to use with the women [41]. However, the GPs in the current sample did not rank resources to use with women highly. This suggests that while GPs in the current sample and women with a history of HDP agree that similar strategies would assist in ensuring women are provided with best practice care, multiple strategies are likely required to achieve this.

While the current sample was from one health district in NSW, Australia, the HNELHD is the only district in NSW to include a major metropolitan centre, several large regional centres, and many smaller rural and remote communities within its borders. Therefore, the district is representative of a variety of regions across Australia and results from this survey are consistent with other Australian surveys [16,29]. However, although an analysis was undertaken to determine differences in current practices, barriers, facilitators, and potential strategies to improve CVD prevention amongst women with HDP by geographical location and age, interpretation of the findings has taken into consideration the small sample size of GPs and the large metropolitan or regional geographical locations of GPs and women with a history of HDP. Although the survey of GPs has a small sample size, it is consistent with surveys conducted with Australian GPs [25] where low response rates have been consistently documented [42]. The surveys were also impacted by the COVID-19 pandemic, in particular the survey of GPs, which took place during the nationwide mandatory vaccination program rolled-out in key health care settings, including primary health care. This likely had a negative effect on the response rate of this survey. This and the predominantly female sample of GPs limits the external validity of results. Recruiting a larger and more representative sample of GPs and women with a history of HDP, however, is essential to guide future research. Additionally, a majority of the participants in the history of HDP survey experienced HDP within the past two years, allowing us to identify the current practice. However, due to the timing of the survey, the practice reported may not be reflective of the usual practice due to the effect that COVID-19 lockdowns may have had on health care provisions (e.g., ability to make a GP appointment and prioritization of preventative care-focused appointments). The current study did not explore whether the care received by women differed by type and/or severity of HDP. As this may influence CVD risk and, therefore, have the potential to influence the provision of CVD prevention, future research should explore these differences. Lastly, the survey did not consider other pregnancy complications, such as gestational diabetes; therefore, authors could not determine whether postpartum care differed with the presence of additional health conditions.

## 5. Conclusions

While it seems that the HNELHD women with a history of HDP are receiving advice consistent with ISSHP and SOMANZ guidelines for traditional CVD risk markers, they are less likely to receive CVD preventative care for female-specific risk factors or lifestyle CVD risk factors. Additionally, while GPs and women with a history of HDP identified several barriers to providing or accessing CVD preventative care (e.g., lack of skills and training, insufficient environmental support and resources for CVD prevention post-HDP, and difficulty obtaining an appointment), these did not always align with strategies that they believed would improve the provision of CVD preventive care. 

## Figures and Tables

**Table 1:** Demographic characteristics of general practitioners and women with a history of hypertensive disorders of pregnancy from Hunter New England Health Local Health District who completed a questionnaire regarding preventive cardiovascular care. Figures shown as mean (SD) or number (%) as appropriate.

	General Practitioners (*n* = 35)	Women with a History of HDP (*n* = 105)
Variables	Data	
Age (years)	Mean (SD)	
	45 (11.8)	31 (4.9)
Demographic characteristics	Total *n* (%)	
Gender		
Male	5 (14.3)	NA
Female	30 (85.7)	105 (100)
Non-Binary	0 (0)	0 (0)
Other	0 (0)	0 (0)
* Practice or residential area [33]		
Metropolitan	31 (60.0)	70 (66.7)
Regional/rural	14 (40.0)	35 (33.3)
Most recent HDP diagnosis		
2017	NA	10 (9.5)
2018	NA	13 (12.4)
2019	NA	14 (13.3)
2020	NA	29 (27.6)
2021–2022	NA	39 (37.1)
General practitioners’ main practice		
Sole practitioner	1 (2.9)	NA
Small group practice (≤5 clinicians)	11 (31.4)	NA
Large group practice (≥6 clinicians)	22 (62.9)	NA
Hospital-based	1 (2.9)	NA

NA: not applicable. HDP: hypertensive disorders of pregnancy. * The Hunter New England Local Health District covers regional, rural, and remote communities. Greater Newcastle and the Lower Mid North Coast sectors cover metropolitan and regional communities, whereas rural communities are covered by the Lower Hunter/Hunter Valley, Peel, Tablelands, and Mehi sectors. Within these rural sectors also sit remote communities [33]. Number of GPs and women in each sector included: Greater Newcastle *n* = 21 (60% GPs) and *n* = 70 (66.7% women), Lower Hunter/Hunter Valley *n* = 8 (22.9% GPs) and *n* = 30 (28.6% women), Lower Mid North Coast *n* = 4 (11.4% GPs), Peel *n* = 1 (2.9% GPs) and *n* = 5 (4.8% women), Tablelands *n* = 1 (2.9% GP), and there were no GPs or women with HDP history in the Mehi sector.

**Table 2:** General practitioners (*n* = 35) self-reported frequency of providing cardiovascular assessment and management of risk markers, lifestyle risk factors, and medical risk factors for women following hypertensive disorders of pregnancy.

**CVD Risk Marker Measurement** **Practices of Women with HDP History** **(*n* = 34)**	**Every 2–5 Years**	**Annually**	**Bi-Annually (or More Frequently)**
	***n* (%)**		
Blood pressure	1 (2.9)	5 (14.7)	28 (82.4)
Blood lipids *	9 (26.5)	23 (67.6)	2 (5.9)
Blood glucose and/or insulin	10 (29.4)	23 (67.6)	1 (2.9)
**Lifestyle risk factor measurement (*n* = 35)**	**Never/occasionally**	**Sometimes**	**Often/always**
	***n* (%)**		
Diet	6 (17.1)	9 (25.7)	20 (57.1)
Physical activity/sedentary behaviour	3 (8.6)	6 (17.1)	26 (74.3)
Smoking status	1 (2.9)	1 (2.9)	33 (94.3)
Alcohol consumption	2 (5.7)	4 (11.4)	29 (82.9)
Body weight/overweight or obesity	1 (2.9)	5 (14.3)	29 (82.9)
Sleep habits	10 (28.6)	16 (45.7)	9 (25.7)
Stress/mental health	6 (17.1)	6 (17.1)	23 (65.7)
**Medical History Risks (*n* = 33)**	**Never/occasionally**	**Sometimes**	**Often/always**
	***n* (%)**		
Family history of heart disease	1 (3.0)	1 (3.0)	31 (93.9)
Familial hypercholesterolemia	0 (0.0)	3 (9.1)	30 (90.1)
High blood pressure	0 (0.0)	0 (0.0)	33 (100.0)
High blood glucose levels	0 (0.0)	0 (0.0)	33 (100.0)
High plasma lipid levels	0 (0.0)	0 (0.0)	33 (100.0)
Overweight or obesity	0 (0.0)	2 (6.1)	31 (93.9)
Preeclampsia	4 (12.1)	11 (33.3)	18 (54.5)
Gestational hypertension	4 (12.1)	9 (27.3)	20 (60.6)
Gestational diabetes	1 (3.0)	9 (27.3)	23 (69.7)
Pre-term birth	13 (39.4)	11 (33.3)	9 (27.3)
Small for gestational age	15 (45.5)	9 (27.3)	9 (27.3)
Polycystic ovarian syndrome	8 (24.2)	10 (30.3)	15 (45.5)

CVD: cardiovascular disease. HDP: hypertensive disorders of pregnancy. * Blood lipids include total cholesterol, high-density lipoproteins (HDL), low-density lipoproteins (LDL), and triglycerides.

**Table 3:** Women with a history of hypertensive disorder of pregnancy (*n* = 105) and the awareness of cardiovascular risks, risk markers, and lifestyle risk factors assessment by a health professional.

CVD Risk	Total *n* (%)
Awareness of CVD risk	29 (17%)
CVD risk marker and lifestyle CVD risk factor assessment by a health professional	
CVD * risk markers	
Obstetric history	76 (72.4)
Blood pressure	87 (82.9)
Blood lipids	26 (24.8)
Blood glucose and/or insulin	42 (40.0)
Lifestyle CVD * risk factors	
Diet	54 (51.4)
Physical activity	60 (57.1)
Smoking status	51 (48.6)
Alcohol consumption	53 (50.5)
Sleep habits	47 (44.8)
Body weight/overweight or obesity	44 (41.9)
Stress/mental health	84 (80.0)

* CVD: cardiovascular disease. Women post-HDP were asked whether general practitioners, midwives, and/or obstetricians assessed CVD risk markers after having HDP, whereas women after HDP were asked whether general practitioners, midwives, obstetricians, and allied health professionals assessed lifestyle CVD risk factors after HDP. This table demonstrates the number of women who had at least one health professional assess these CVD risk markers.

**Table 4:** Mean scores for Theoretical Domains Framework barriers and facilitators reported by general practitioners (*n* = 33), where a higher mean score indicates more agreement with the statement corresponding to each domain.

Barrier/Facilitators	Mean *	SD
Knowledge (I know what my responsibilities are regarding assessment and management of lifestyle risk factors in women following hypertensive pregnancies.)	4.6	0.5
Belief about consequences (I believe that providing advice for managing lifestyle risk factors will positively benefit women following hypertensive pregnancies.)	4.6	0.6
Optimism (I do not believe that anything will prevent me from assessing and managing lifestyle risk factors when seeing a woman following a hypertensive pregnancy.)	4.4	1.0
Behavioural regulation (I have a detailed plan of how and when I will assess lifestyle risk factors when I see women after hypertensive pregnancies.)	4.1	0.6
Social influences (I am supported and encouraged by my workplace and colleagues to routinely offer assessment and management of lifestyle risk factors as part of usual care for women post-hypertensive pregnancies.)	4.0	0.9
Belief about capabilities (I have the confidence to advise women with a history of hypertensive pregnancies of their increased CVD risk.)	3.9	0.9
Social/professional role and identity (It is my role to assess and manage lifestyle risk factors among women following hypertensive pregnancies.)	3.9	0.9
Goals (Compared to other tasks, assessing and managing lifestyle risk factors in women following a hypertensive pregnancy is a high priority.)	3.7	0.9
Reinforcement (It is important to reiterate to women who have had a hypertensive pregnancy that they will have a life-long increased risk of cardiovascular disease.)	3.6	1.0
Intentions (I have strong intentions to assess and manage lifestyle risk factors in women following hypertensive pregnancies.)	3.6	0.8
Memory, attention, and decision processes (Assessing and managing lifestyle risk factors in women following hypertensive pregnancies is something I do not regularly do.)	3.5	1.2
Emotion (I can discuss the benefits of managing lifestyle risk factors with women following hypertensive pregnancies even if they are not contemplating behaviour change.)	3.5	0.8
Skills (I have received training in how to assess and manage lifestyle risk factors among women following hypertensive pregnancies.)	3.4	1.1
Environmental context and resources (In my practice, there are sufficient resources and training enabling me to assess and manage lifestyle risk factors among women following hypertensive pregnancies.)	3.1	1.1

* Constructs are reported on a 5-point Likert scale ranging from 1 = strongly disagree to 5 = strongly agree. Table adapted from Grady et al., 2018 [34]. CVD: cardiovascular disease. CVD care includes the assessment and management of lifestyle and medical risk factors for CVD in women following an HDP.

**Table 5:** Additional barriers to assessing and managing lifestyle risk factors for women after hypertensive disorders of pregnancy identified by general practitioners through thematic analysis.

Themes	Quotes
Lack of time	“Often there are more pressing matters and higher priority tasks during patient appointments.”
	“Limited opportunity, time constraints and competing clinical priorities.”
Lack of general practitioners’ knowledge and training	“I feel confident in considering the common risk factors (e.g., smoking, diabetes, hypertension), but admittedly I haven’t always thought to ask about hypertensive disorders in pregnancy.”
	“Not aware of the long-term implications of this condition.”
Lack of patient awareness and motivation	“Patients are not aware of the importance of follow-up, or long-term risks. Usually, they have not been educated about this during obstetric care or only mentioned in passing.”
	“Often women are unaware of any ongoing risk following pregnancy, which can make engaging them in lifestyle changes postpartum challenging as they are already overwhelmed with lots of other information.”
Lack of patients’ obstetric history	“Identifying who has had a hypertensive disorder of pregnancy can be challenging. It is not documented as a current problem and often does not make it out of the hospital discharge summary to the patient’s file.”
	“Often women will not disclose their history of hypertensive disorder of pregnancy.”

**Table 6:** Women with a history of hypertensive disorders of pregnancy (*n* = 105) self-reported barriers experienced when accessing cardiovascular prevention care from general practitioners.

**^$^ Barriers reported by women with past HDP to accessing GP services in general**	***n* (%)**
Difficulty booking/obtaining an appointment	59 (56.2)
Time	38 (36.2)
Cost	36 (34.3)
Lack of childcare facilities	20 (19.1)
Inconvenient opening hours	19 (18.1)
Other	11 (10.5)
Transport	2 (1.9)
Lack of understanding of resources	1 (1.0)
Language/cultural barrier	0 (0.0)
**Barriers to accessing cardiovascular disease care after HDP**	***n* (%)**
Number of women satisfied by the treatment provided by a GP *	83 (79.1)
Number of women who feel they have enough time to discuss heart health strategies with a GP *	50 (47.6)
Number of women who have been directed to resources for heart disease prevention by a GP *	1 (1.0)

* GP: general practitioner. HDP: hypertensive disorders of pregnancy. ^$^ Participants could select more than one barrier to accessing GP services, and therefore, percentages within the table do not add to 100%.

**Table 7:** Level of support women with a history of hypertensive disorders of pregnancy (*n* = 105) felt from their general practitioner in making changes to lifestyle CVD risk factors.

Lifestyle CVD ^@^ Risk Factors	Very Supported	Somewhat Supported	Neutral	Not Very Supported	Not at All Supported	Not Applicable to Them
	*n* (%)					
Diet	32 (30.5)	25 (23.8)	26 (24.8)	10 (9.5)	3 (2.9)	9 (8.6)
Physical activity	29 (27.6)	26 (24.8)	25 (23.8)	12 (11.4)	3 (2.9)	10 (9.5)
Smoking status	16 (15.2)	5 (4.8)	8 (7.6)	3 (2.9)	3 (2.9)	70 (66.7)
Alcohol consumption	19 (18.1)	7 (6.7)	11 (10.5)	5 (4.8)	3 (2.9)	60 (57.1)
Body weight/overweight or obesity	33 (31.4)	19 (18.1)	26 (24.8)	9 (8.6)	4 (3.8)	14 (13.3)
Sleep habits	24 (22.9)	18 (17.1)	26 (24.8)	15 (14.3)	5 (4.8)	17 (16.2)
Stress/mental health	43 (41.0)	29 (27.6)	16 (15.2)	6 (5.7)	3 (2.9)	8 (7.6)

^@^ CVD: cardiovascular disease.

**Table 8:** Percentage of general practitioners and women who rated the below strategies in their top three preferred strategies to improve the cardiovascular preventive care in primary care.

Strategies	General Practitioners *n* (%)	Women with a History of HDP *n* (%)
Improved communication to women of the long-term effects of a high blood pressure problem during pregnancy	23 (69.7)	62 (59.1)
Improved communication between all health professionals (e.g., GP and hospital systems)	22 (66.7)	58 (55.2)
Improved systems within practices that alert GPs to women who have had HDP during/prior to consultation ^^^	17 (51.5)	NA
GPs referring women to see another health professional for further advice to reduce CVD (e.g., a Cardiologist, Physiotherapist, Dietitian)	2 (6.1)	52 (49.5)
GPs to use resources within a consultation to provide appropriate assessment and advice on heart disease	8 (24.2)	39 (37.1)
GPs to use a checklist of questions to ask women after HDP during their first postpartum appointment ^^^	NA	30 (28.6)
Professional development for GPs in the areas of hypertensive disorders of pregnancy and CVD prevention ^^^	12 (33.3)	NA
GPs to provide resources and strategies (printed or digital) for women to take home after consultations.	10 (30.3)	26 (24.8)
A GP to provide women with pre-pregnancy planning advice, discussing potential pregnancy complications ^^^	NA	21 (20.0)
Improved communication from GPs to women regarding their ongoing health care ^^^	NA	15 (14.3)
Include training in this area as part of the medical degree for GPs ^^^	2 (6.1)	NA

GP: general practitioner. ^^^ These questions were only asked in either the GP survey or the survey of women after HDP, but not both.

**Table 9:** Additional strategies that would support women after hypertensive disorders of pregnancy to obtain cardiovascular disease assessment and management from general practitioners, identified through thematic analysis.

Themes	Quotes
More support and/or acknowledgement of obstetric history	“Just basic information would be a great start; I had no idea until I found this survey.”
	“Ask what happened during your pregnancy. If asked about my preeclampsia, I may have been told about heart issues later.”
	“Having had GDM as well, I feel like the follow-up for that has been a lot more involved and flagged in the GPs notes on my file. It does not seem as though having preeclampsia triggered the same level of ongoing support or action at all.”
Attending more than one postpartum appointment and/or a heart heath specific appointment	“Requesting a postpartum appointment (outside of the 6-week check) to specifically discuss heart health.”
	“I’ve never had a follow-up with my heart with any specialists after having both gestational hypertension and preeclampsia.”
	“More postpartum check-ups more than the 6-week one.”
Being send regular reminders to follow-up with the GP	“Reminder text messages for routine care and tests required if long-term follow-up required.”
	“Maybe send a recall letter every year for a heart check.”
	“Reminders to come in to assess heart health.”

## Data Availability

The original contributions presented in the study are included in the article; further inquiries can be directed to the corresponding author.
